# Concerns regarding calculation of fatality rate of Chinese samples in COVID‐19 meta‐analysis

**DOI:** 10.1002/jmv.25799

**Published:** 2020-04-10

**Authors:** Matthew J. Kempton

**Affiliations:** ^1^ Institute of Psychiatry, Psychology and Neuroscience King's College London London United Kingdom

Dear Editor,

I read with interest the meta‐analysis of patient clinical characteristics and fatality rates in COVID‐19 from Chinese samples by Li et al.^1^ I was surprised by the fatality rate of 7% which is much than higher than the value of 1.4% reported in a large study of Chinese patients by Guan et al.^2^

Li et al do not include Guan et al in their meta‐analysis of fatality rates and no reason is given for its omission. This is a concern, as the study by Guan et al is twice as large as all the other studies reporting fatality rates combined, and would significantly reduce the overall pooled fatality rate.
